# Mosaicism in *BRPF1*-Related Neurodevelopmental Disorder: Report of Two Sisters and Literature Review

**DOI:** 10.1155/2023/1692422

**Published:** 2023-11-01

**Authors:** Khaliunaa Bayanbold, Georgianne Younger, Benjamin Darbro, Alpa Sidhu

**Affiliations:** ^1^Free Radical Radiation Biology, Department of Radiation Oncology, University of Iowa Hospitals and Clinics, Iowa City, IA, USA; ^2^Division of Medical Genetics and Genomics, The Stead Family Department of Pediatrics, University of Iowa Hospitals and Clinics, Iowa City, IA, USA

## Abstract

Bromodomain and PHD finger containing 1 (*BRPF1*)-related neurodevelopmental disorder is characterized by intellectual disability, developmental delay, hypotonia, dysmorphic facial features, ptosis, and blepharophimosis. Both *de novo* and inherited pathogenic variants have been previously reported in association with this disorder. We report two affected female siblings with a novel variant in *BRPF1* c.2420_2433del (p.Q807Lfs*∗*27) identified through whole-exome sequencing. Their history of mild intellectual disability, speech delay, attention deficient hyperactivity disorder (ADHD), and ptosis align with the features previously reported in the literature. The absence of the *BRPF1* variant in parental buccal samples provides evidence of a *de novo* frameshift pathogenic variant, most likely as a result of parental gonadal mosaicism, which has not been previously reported. The frameshift pathogenic variant reported here lends further support to haploinsufficiency as the underlying mechanism of disease. We review the literature, compare the clinical features seen in our patients with others reported, and explore the possibility of genotype-phenotype correlation based on the location of pathogenic variants in *BRPF1*. Our study helps to summarize available knowledge and report the first case of a *de novo* frameshift pathogenic variant in *BRPF1* in two siblings with this neurodevelopmental disorder.

## 1. Introduction

*BRPF1*-related neurodevelopmental disorder was first described by Mattioli et al. [1] and Yan et al. [2]. Pathogenic variants in the *BRPF1* gene cause an intellectual developmental disorder with dysmorphic facies and ptosis (IDDDFP). This disorder is inherited in an autosomal dominant manner and is characterized by delayed psychomotor development, language delay, intellectual disability, and dysmorphic facial features that include ptosis and blepharophimosis (OMIM 617333) [1, 2]. Other reported clinical features include hypotonia, seizures, short stature, and microcephaly [1, 2]. Two case reports have described additional features that may be associated with IDDDFP, including colobomas, facial nerve palsy, severe hypoplasia of the corpus callosum, and sudden unexplained death in childhood [3, 4]. Maternal mosaicism has been reported in one family with the unaffected mother identified to have 7% mosaicism in peripheral blood [2]. Here, we present the first case of two affected female siblings with mild intellectual disability, speech delay, ADHD, and ptosis with a *de novo* frameshift pathogenic variant in the *BRPF1* gene due to suspected parental gonadal mosaicism. In our study, we review the literature to summarize the clinical features of this neurodevelopmental disorder, expand the phenotype, and explore the possibility of genotype-phenotype correlation based on the location of pathogenic variants in the *BRPF1* gene.

## 2. Case Report

**Figure 1:**
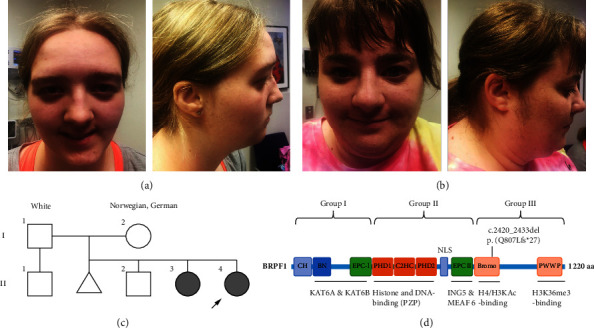
(a) Facial appearance of the proband at 23 years of age. Note the broad nasal root, bilateral mild ptosis, down slanted palpebral fissures, and macrocephaly. (b) Facial appearance of the sister at 29 years of age. Note the broad nasal root, posteriorly rotated ears, and macrocephaly. (c) Two-generation pedigree. Shaded symbols designate affected individuals with the heterozygous pathogenic *BRPF1* variant c.2420_2433del (p.Q807Lfs*∗*27). The arrow indicates the proband. (d) Schematic representation of BRPF1 protein showing different domains and the location of the pathogenic variant identified in our patients. Based on functional domain and location of pathogenic variant, three groups were created for genotype-phenotype correlation study. The figure is adapted from [2].

The proband (designated patient II-4 in Figure 1(c)) is a 23-year-old female, born full term to nonconsanguineous parents, after an uncomplicated pregnancy without any postnatal complications. Parental race was reported as White with maternal ancestry specified as Norwegian and German. Early developmental milestones were reported to be delayed primarily for speech and communication skills, for which she received speech therapy. The proband attended a mix of regular and special education classes throughout school and completed her high school diploma. She was diagnosed with ADHD and mild intellectual disability (full-scale IQ 65). Upon physical examination in the clinic, she was found to have macrocephaly, down-slanted palpebral fissures, retrognathia, mild bilateral ptosis (Figure 1(a)), and a normal gait. Brain imaging was not performed.

Initial genetic evaluations were nondiagnostic. These included *FMR1* repeat (28, 29 repeats) and chromosomal microarray analysis which identified a 241 kilobase deletion classified as a variant of uncertain significance GRCh37/hg19 5q31.3 (chr5: 142256830-142498252)x1. This copy number variant involves a partial deletion of the *ARHGAP26* gene and does not overlap any currently known constitutional haploinsufficient genes or genomic regions.

The proband's older sister (designated patient II-3 in Figure 1(c)) is a 29-year-old female with a similar clinical history. Her pregnancy and birth history were unremarkable. She had developmental delays primarily in speech and communication and attended both regular and special education classes to receive her high school diploma. She was diagnosed with ADHD and mild intellectual disability (full-scale IQ 70). She wears corrective lenses for myopia and has a history of hypertension and hyperlipidemia. Physical examination findings were remarkable for macrocephaly, posteriorly rotated ears (Figure 1(b)), and a wide-based gait. A two-generation pedigree is depicted in Figure 1(c).

Whole-exome sequencing (WES) analysis was performed on DNA extracted from peripheral blood samples for the proband and her sister. WES analysis identified a heterozygous pathogenic variant in *BRPF1* c.2420_2433del (p.Q807Lfs*∗*27) in both sisters. The variant was classified as pathogenic due to it being a de novo, null variant that was absent from gnomAD, appropriately segregated with the disease, and is within a gene known to cause a similar disease phenotype. The variant was not identified in parental buccal samples, strongly suggesting gonadal mosaicism in one of the parents. We performed visual analysis of the aligned sequencing read data from the parents' buccal samples to determine the parent-of-origin of the allele on which the *BRPF1* pathogenic variant occurred. However, there were no nearby informative single-nucleotide polymorphisms (SNPs) within the range applicable to short-read sequencing data (150 bp paired-end reads). Hence, parent-of-origin could not be determined.

## 3. Materials and Methods

### 3.1. Chromosomal Microarray

Chromosomal oligonucleotide microarray and SNP analysis were performed using an Affymetrix CytoScanHD hg19 (NCBI build 37) whole-genome array consisting of 1.9 million nonpolymorphic markers and 750,000 SNP probes, with an average probe spacing of about 1.2 kb. Affymetrix ChAS software (Affymetrix, version 1.2.2) and Nexus Copy Number (BioDiscovery, version 7) software were applied to process and analyze the data.

### 3.2. Whole-Exome Sequencing

Whole-exome sequencing was performed through GeneDx testing laboratory, Gaithersburg, MD, USA.

### 3.3. Informed Consent

Informed consent was obtained from the family for analysis of WES data and publication.

## 4. Discussion

Pathogenic variants in *BRPF1* have been associated with neurodevelopmental features known as intellectual developmental disorder with dysmorphic facies and ptosis (IDDDFP) (OMIM 617333) [1, 2]. Common clinical features of IDDDFP include intellectual disability (ID), global developmental delay, hypotonia, facial dysmorphisms, ptosis, and/or blepharophimosis. Less frequently reported clinical features include hand and foot anomalies, brain anomalies, microcephaly, behavioral anomalies, growth retardation, and seizures [1–9]. Maternal mosaicism has been previously reported in Yan et al. [2]. Here, we report on two siblings with a *de novo* frameshift pathogenic variant in the *BRPF1* gene, c.2420_2433del (p.Q807Lfs*∗*27). Parental testing on buccal samples did not identify this variant, suggesting gonadal mosaicism. This has important reproductive implications for families in which *BRPF1* variants appear to occur *de novo* [10]. Genetic counseling regarding the possibility of gonadal mosaicism should be considered for family members of an individual with IDDDFP. Analysis of sibling and parent WES data was unable to identify the parent-of-origin of the variant, so we are unable to draw conclusions regarding the type of gonadogenesis involved.

Previous studies have identified pathogenic variants throughout the *BRPF1* gene and, therefore, within important functional protein domains. The BRPF1 protein is a large multivalent chromatin reader composed of multiple nucleosome-binding modules. These include the BRPF1 specific N-terminal (BN) at the N-terminus, enhancer of polycomb (EPC) like motif 1 (EPC-1), plant homeodomain (PHD)-zinc-knuckle-PHD (PZP) module, nuclear localization signal (NLS), EPC-II, a bromodomain, and a C-terminal proline-tryptophan-tryptophan-proline (PWWP) domain [9, 11–14]. The EPC-I and BN domains are required for binding to the MYST domain of KAT6A or KAT6B, whereas the PZP domain functions by recognizing histone H3 tails and associating them with DNA [9, 15–17]. EPC-II interacts with two accessory proteins, ING5 and MEAF6, while the bromodomain binds to acetyl-lysine in histone H4 and H3 (H4/H3KAc) [18–21]. The PWWP domain is required for BRPF1 to bind condensed chromatin and recognize trimethylated K36 of histone H3 (H3K36me3) [22, 23].

Given the different functional roles of the BRPF1 protein domains, we reviewed the literature and divided all reported patients into three groups by variant location to assess for genotype-phenotype relationships. Group I includes patients harboring a pathogenic *BRPF1* variant in the region of the KAT6/KAT6B binding domain. Group II includes patients with a pathogenic *BRPF1* variant in the PZP module or the region that interacts with ING5 and MEAF6. Lastly, Group III includes those with a pathogenic variant in the bromodomain or PWWP domain that interacts with H4/H3KAc and H3K36me3 (Figure 1(d)).  Table 1 shows the genotype-phenotype correlation among the three groups [1–4, 6–9]. Our siblings were included in Group III. Additional details can be found in the Supplemental Tables 1Athrough 1D. Those without reported phenotypic information were not included in our analysis [24–26]. Three patients from Mattioli et al. [1] and one patient from Abarca-Barriga et al. [5] were also excluded as they have larger multigene deletions.

Consistent with previous reports, common clinical features seen across all three groups include neurological features and facial dysmorphism [1–4, 6–9]. The majority of patients in all groups had delays in walking, speech delay, and intellectual disability (ID) (Table 1). We were unable to draw conclusions between variant location and the degree of ID (Supplemental Tables 1Athrough 1D). Similarly, dysmorphic facial features of flat facial profile, down-slanted palpebral fissures, broad nasal root, round face, and hypertelorism were seen among all groups. Less frequent features observed include seizures, short stature, feeding difficulties, and brain anomalies. Despite the similarities described previously, some differences between groups were uncovered. Individuals in Group I (KAT6A/KAT6B binding) and Group II (PZP module binding) were reported to have microcephaly (25% and 18%, respectively) that was not observed in Group III. While the numbers are small, 75% of patients in Group III (bromodomain) were found to be macrocephalic and were only reported in 13% of patients in Group I. Ptosis and blepharophimosis, common features of IDDDFP, were observed in most individuals from Groups I (62% and 67%) and II (74% and 56%). These features were less common in Group III (38% and 25%). Of our patients, only one had mild ptosis and neither had blepharophimosis. Most patients from Group II displayed feeding difficulty (67%) and short stature (50%) compared to Group I (25% and 11%) and Group III (25% and 20%). Behavioral anomalies were more prevalent in Groups I and III (75%) compared to Group II (17%).

Overall, *BRPF1* is a central player in chromatin modification by regulating histone acetyltransferases and is involved in stem cell renewal, hematopoiesis, embryo survival, head patterning, and brain development [13, 27–31]. Furthermore, *BRPF1* is abundantly expressed in testes and spermatogonia indicating important reproductive implications of the *BRPF1* gene [32]. Pathogenic variants in *BRPF1* are known to cause aberrant histone acetylation and intellectual disability disorders [1, 2, 9, 31]. The greater prevalence of microcephaly, ptosis, and blepharophimosis in Group I compared to Group III may reflect the importance of BRPF1 and KAT6A/B interaction considering pathogenic variants in KAT6A/B are also known to cause similar clinical features [33–38]. Specific mutation types in Group III, which impact the bromodomain, conserved structural motifs, and involved in the recognition of acetylated histones, may be attributable to *BRPF1* haploinsufficiency. Although we observed some trends between genotype and phenotype among the individuals reported in the literature, it is hard to draw any broad conclusions due to the importance of each functional domain of BRPF1 and their interaction with both histones and DNA for epigenetic regulation. There are also limitations to the phenotypic data as not all reports described the same features included in Table 1, and some groups had as few as 3 patients, or as many as 19, included in each feature category.

In summary, we report on the first case of a *de novo* frameshift pathogenic variant in the *BRPF1* gene in two affected siblings, due to suspected parental gonadal mosaicism. Clinical features seen in our patients include mild intellectual disability, speech delay, and facial dysmorphisms, which overlap with what has been previously reported. Macrocephaly and ADHD, rarely reported features of the disorder, were seen in our patients. Additional clinical and functional studies are needed to further explore the relationship between *BRPF1* pathogenic variants in KAT6A/B-binding sites and microcephaly, PZP modules and feeding issues or short stature, and bromodomains and macrocephaly. An extensive review of the patients reported in the literature did not show any conclusive genotype-phenotype correlation in the three groups based on variants and domains of the protein.

## Figures and Tables

**Table 1:** Clinical features of patients grouped by *BRPF1* pathogenic variant location.

	Group I *N* (%)	Group II *N* (%)	Group III *N* (%)	Our case proband	Our case sibling
Pathogenic variant (inherited/*de novo*)	Inherited 7 *De novo* 6	Inherited 5 *De novo* 10	Inherited 1 *De novo* 7	*De novo*	*De novo*
Age at diagnosis	3–61 Y	1–34 Y	2–28 Y	23 Y	28 Y
Sex	7M, 6F	10M, 8F	3M, 4F	F	F
Craniofacial features					
Flat facial profile	2/8 (25)	3/9 (33)	4/7 (57)	−	−
DSPF	7/13 (54)	6/15 (40)	4/7 (57)	+	−
Broad nasal root	3/7 (43)	7/13 (54)	5/6 (83)	+	+
Round face	4/9 (44)	8/15 (53)	3/8 (38)	−	−
Hypertelorism	6/10 (60)	7/13 (54)	5/7 (71)	−	−
Head circumference					
Macrocephaly	1/8 (13)	0/11 (0)	3/4 (75)	+	+
Microcephaly	2/8 (25)	2/11 (18)	0/3 (0)	−	−
Neurological features					
Delay in walking	8/10 (80)	14/16 (88)	5/8 (63)	−	−
Speech delay	7/10 (70)	14/16 (88)	8/8 (100)	+	+
Intellectual disability	7/13 (54)	14/16 (88)	8/8 (100)	Mild	Mild
Behavioral anomalies	6/8 (75)	2/12 (17)	3/4 (75)	ADHD	ADHD
Seizures	2/13 (15)	5/19 (26)	2/8 (25)	−	−
Brain abnormalities	2/4 (50)	4/11 (36)	3/5 (60)	NA	NA
Eye					
Ptosis	8/13 (62)	14/19 (74)	3/8 (38)	+	−
Blepharophimosis	8/12 (67)	10/18 (56)	2/8 (25)	−	−
Musculoskeletal anomalies					
Hand	3/8 (38)	6/11 (55)	2/5 (40)	−	−
Foot	1/8 (13)	3/10 (30)	0/3 (0)	−	−
Growth					
Feeding difficulty	2/8 (25)	8/12 (67)	1/4 (25)	−	−
Short stature	1/9 (11)	6/12 (50)	1/5 (20)	−	−

ADHD: attention deficit hyperactivity disorder, DSPF: down-slanted palpebral fissures, M: male, F: female, NA: information not available, Y: years, +: feature present, −: feature absent.

## Data Availability

Data sharing is not applicable to this article as no new data were created or analysed in this study.

## Supplementary Materials

Supplementary Materials Table 1: A) Distribution of reported cases of *BRPF1*-related disorder into three groups based on domain. B) Phenotype of patients in Group I. C) Phenotype of patients in Group II. D) Phenotype of patients in Group III.
