# Supplementary material for: Mosaicism in BRPF1-Related Neurodevelopmental Disorder: Report of Two Sisters and Literature Review
Source: Case Rep Genet. 2023 Nov 1;2023:1692422. doi: 10.1155/2023/1692422 (PMC10632058; doi:10.1155/2023/1692422)
Supplement: Supplementary Materials — Table 1: A) Distribution of reported cases of BRPF1-related disorder into three groups based on domain. B) Phenotype of patients in Group I. C) Phenotype of patients in Group II. D) Phenotype of patients in Group III. [file 1692422.f1.docx]

**Supplemental Table 1A : Distribution of reported cases in the literature into three groups**

|  | **Group I:** KAT6A/KAT6B binding | **Group II:** PZP module | **Group III:** Bromo and PWWP domain |
| --- | --- | --- | --- |
| **Mattioli et al., 2017** | c.104dupA (p.Tyr35*) | c.1052_1053del (p.Val351Glyfs*8) | c.2982C>G (p.Tyr994*) |
|  | c.567delT (p.Asp190Metfs*24) | c.1165T>C (p.Cys389Arg) |  |
| **Yan et al., 2017** | c.362_363delAG (p.Glu121Glyfs*2) | c.942_955del (p.Trp315Leufs*26) | c.2497C>T (p.Arg833*) |
|  |  | c.1108C>T (p.Pro370Ser) | c.2915dup (p.Met973Asnfs*24) |
|  |  | c.1363C>T (p.Arg455*) | c.3298C>T (p.Arg1100*) |
|  |  | c.1688_1689del (p.His563Profs*8) |  |
|  |  | c.1883_1886dup (p.Gln629Hisfs*34) |  |
| **Yan et al., 2020** | c.227C> T (p.Pro76Leu) | c.953G>A (p.Arg318His) | c.3459_3461del CTT (p.Phe1154del) |
|  | c.286C>T (p.Gln96*) | c.1229A >G (p.His410Arg) |  |
|  | c.558_570del13 ins11 (p.Asp187Glyfs*29) | c.1300delA (p.Thr434Profs*61) |  |
|  | c.883_884del (p.Met295Valfs*17) | c.1420dupG (p.Glu474Glyfs*3) |  |
|  |  | c.1622_ 1626dup (p.Tyr543Thrfs*6) |  |
| **Pode-Shakked et al., 2019** | c.556C>T (p.Q186*) |  |  |
| **Naseer et al., 2020** |  | c.1054G>C (p.Val352Leu) |  |
| **Demeulenaere et al., 2019** | c.655G > T (p.Glu219*) |  |  |
| **Keyman et al., 2020** |  | c.1182_1183delAG (p.Ala396LeufsTer69) |  |
| **Souza et al., 2022** |  | c.964C>T (p.Gln322*) |  |
| **Our Case** |  |  | c.2420_2433del (p.Q807Lfs*27) |
| **#Patients** | **13** | **20** | **8** |

**Supplemental Table 1B: Phenotype of patients in Group I**

| Features Group I | c.104dupA (p.Tyr35*) | c.567delT(p.Asp190Metfs*24) | c.227C> T (p. Pro76Leu) | c.286C> T (p. Gln96*) | c.558_570del13 ins11 (p.Asp187Glyfs*29) | c.883_884del  (p.Met295Valfs*17) | | c.362_363delAG (p.Glu121Glyfs*2) | c.556C>T (p.Q186*) | | | | c.655G > T (p.Glu219*) |
| --- | --- | --- | --- | --- | --- | --- | --- | --- | --- | --- | --- | --- | --- |
| Pathogenic Variant  (inherited/*de novo*) | Inherited | *De novo* | Inherited | *De novo* | *De novo* | Inherited | *De novo* | *De novo* | Inherited | | | | *De novo* |
| Age at diagnosis | 12Y | 3Y | 3Y 9M | 5Y 2M | 12Y 7M | 3Y 10M | 34Y | 6Y 5M | 61Y | 42Y | 30Y | 24Y | 3Y 9M |
| Sex | M | M | M | F | M | F | F | F | F | M | M | F | M |
| Craniofacial features |  |  |  |  |  |  |  |  |  |  |  |  |  |
| Flat facial profile | NR | NR | NR | NR | NR | - | - | + | - | - | - | - | + |
| DSPF | + | + | - | - | - | - | - | - | + | + | + | + | + |
| Broad Nasal Root | NR | NR | - | - | + | - | - | + | NR | NR | NR | NR | + |
| Round face | + | + | - | - | - | - | - | + | NR | NR | NR | NR | + |
| Hypertelorism | NR | NR | - | - | + | - | - | + | NR | + | + | + | + |
| Head circumference |  |  |  |  |  |  |  |  |  |  |  |  |  |
| Macrocephaly | - | - | + | - | - | - | - | NR | NR | NR | NR | NR | - |
| Microcephaly | + | - | - | - | - | - | - | NR | NR | NR | NR | NR | + |
| Developmental delay |  |  |  |  |  |  |  |  | - | + | + | + |  |
| Delay in walking | + | + | - | + | + | + | + | + | - | NR | NR | NR | + |
| Speech delay | + | - | + | + | + | + | - | + | - | NR | NR | NR | + |
| Neurological features |  |  |  |  |  |  |  |  |  |  |  |  |  |
| Intellectual disability | Moderate | Moderate | - | - | - | - | - | + | - | Mild to Moderate | Mild to Moderate | Mild to Moderate | + |
| Behavioral anomalies | Hyperactivity, autism | + | Autism spectrum disorder | Self-stimulating behavior | ADHD | - | - | NR | NR | NR | NR | NR | + |
| Seizures | + | - | - | - | + | - | - | - | - | - | - | - | - |
| Brain abnormalities | NA | NA | - | NA | - | NA | NA | WM hyper-intensity | NA | NA | NA | NA | CC |
| Eye |  |  |  |  |  |  |  |  |  |  |  |  |  |
| Ptosis | + | + | - | - | + | - | - | + | + | + | + | + | - |
| Blepharophimosis | + | + | - | + | - | + | + | NR | - | + | + | + | - |
| Musculoskeletal anomalies |  |  |  |  |  |  |  |  |  |  |  |  |  |
| Hand | CD | CD | - | CD | - | - | - | NR | NR | NR | NR | NR | - |
| Foot | - | - | - | SD | - | - | - | NR | NR | NR | NR | NR | - |
| Growth |  |  |  |  |  |  |  |  |  |  |  |  |  |
| Feeding difficulty | NR | NR | - | - | + | - | - | - | NR | NR | NR | - | + |
| Short stature | + | - |  | - | - | - | - | - | NR | NR | NR | NR | - |

Abbreviations: BD - brachydactyly, CC - corpus callosum abnormality, CD- camptodactyly, DSPF- down slanting palpebral fissures, M-male, F- female, NA - information not available, NR - information not reported, Y - years, M - months, + feature present; – feature absent.

**Supplemental Table 1C: Phenotype of patients in Group II**

| Group II | c.1052_1053del (p.Val351Glyfs*8) | | | | | c.1165T>C (p.Cys389Arg) | | c.1883_1886dup (p.Gln629Hisfs*34) | c.942_955del (p.Trp315Leufs*26) | | | | c.1108C>T (p.Pro370Ser) | c.1363C>T (p.Arg455*) | | c.1688_1689del (p.His563Profs*8) | | c.1054G > C (p.Val352Leu) |
| --- | --- | --- | --- | --- | --- | --- | --- | --- | --- | --- | --- | --- | --- | --- | --- | --- | --- | --- |
| Pathogenic Variant  (inherited/*de novo*) | Inherited | | | | | *De novo* | *De novo* | | |  |  |  | | | *De novo* | *De novo* | *De novo* | |
| Age at diagnosis | 5y 9m | 32y | 34y | 6y10 | 34y | 3y 9m | 12y 6m | | | 13y 3m |  | 10y 6m | | | 8y 3m | 4y | 6y | |
| Sex | M | F | F | F | F | M | M | | | M |  | M | | | F | M | F | |
| Craniofacial features |  |  |  |  |  |  |  | | |  |  |  | | |  |  |  | |
| Flat facial profile | NR | NR | NR | NR | NR | NR | NR | | | - | + | + | | | + | - | NR | |
| DSPF | + | NR | NR | + | + | NR | + | | | + | - | - | | | - | - | NR | |
| Broad Nasal Root | NR | NR | NR | NR | NR | NR | + | | | + | + | + | | | + | - | + | |
| Round face | + | NR | NR | + | + | NR | + | | | + | + | - | | | + | - | NR | |
| Hypertelorism | NR | NR | NR | NR | NR | NR | + | | | + | + | + | | | + | - | + | |
| Head Circumference |  |  |  |  |  |  |  | | |  |  |  | | |  |  |  | |
| Macrocephaly | - | - | - | - | - | - | NR | | | NR | NR | NR | | | NR | NR | - | |
| Microcephaly | - | - | + | - | - | - | NR | | | NR | NR | NR | | | NR | NR | + | |
| Normal | + | + | - | + | + | + | NR | | | NR | NR | NR | | | NR | NR | - | |
| Neurological features |  |  |  |  |  |  |  | | |  |  |  | | |  |  |  | |
| Delay in walking | + | NR | NR | + | NR | + | + | | | + | + | + | | | + | + | + | |
| Speech delay | + | NR | NR | + | NR | + | + | | | + | + | + | | | + | + | + | |
| ID | Mild | Mild | Mild | Mild | Mild | Mild | + | | | + | + | + | | | + | + | + | |
| Behavioral anomalies | - | - | - | Hyperactivity | - | Very shy | NR | | | NR | NR | NR | | | NR | NR | NR | |
| Seizures | - | - | - | - | - | - | + | | | + | + | - | | | + | - | - | |
| Brain abnormalities | ACC | NR | NR | - | NR | - | NA | | | ↓WM | - | - | | | - | NA | Brain Atrophy | |
| Eye |  |  |  |  |  |  |  | | |  |  |  | | |  |  |  | |
| Ptosis | + | + | + | + | + | + | + | | | - | + | - | | | + | + | - | |
| Blepharophimosis | + | + | + | + | + | + | NR | | | - | + | + | | | + | - | - | |
| Musculoskeletal anomalies |  |  |  |  |  |  |  | | |  |  |  | | |  |  |  | |
| Hand | BM, BD | BM,  BD | BM,  BD | BM,  BD | BM,  BD | - | NR | | | NR | NR | NR | | | NR | NR | NR | |
| Foot | CF | - | NR | - | - | CD | NR | | | NR | NR | NR | | | NR | NR | NR | |
| Growth |  |  |  |  |  |  |  | | |  |  |  | | |  |  |  | |
| Feeding difficulty | NR | NR | NR | NR | NR | NR | + | | | + | NR | + | | | - | - | + | |
| Short stature | + | + | + | - | + | - | NR | | | NR | NR | NR | | | NR | NR | NR | |

Abbreviations: BD - brachydactyly, CC - corpus callosum abnormality, CD- camptodactyly, DSPF- down slanting palpebral fissures, M-male, F- female, NA - information not available, NR - information not reported, Y - years, M - months, + feature present; – feature absent.

**Supplemental Table 1D: Phenotype of patients in Group III**

| Group II (continued) | c.953G>A (p.Arg318His) | c.1229A >G (p.His410Arg) | c.1300delA (p.Thr434Profs*61) | c.1420dupG (p.Glu474Glyfs*3) | c.1622_ 1626dup (p.Tyr543Thrfs*6) | c.1182_1183delAG (p.Ala396LeufsTer69) | c.964C>T (p.Gln322*) |
| --- | --- | --- | --- | --- | --- | --- | --- |
| Pathogenic Variant  (inherited/de novo) | De novo | NA | De novo | NA | De novo | De novo | De novo |
| Age at diagnosis | 13M | 12Y | 16M | NA | 6Y | 3Y | 16Y |
| Sex | M | F | F | NA | M | M | M |
| Craniofacial features |  |  |  |  |  |  |  |
| Flat facial profile | - | NR | NR | NA | - | - | - |
| DSPF | - | - | - | NA | - | - | + |
| Broad Nasal Root | - | + | - | NA | - | - | - |
| Round face | - | - | - | NA | - | - | + |
| Hypertelorism | - | - | - | NA | + | - | - |
| Head Circumference |  |  |  |  |  |  |  |
| Macrocephaly | NA | - | - | NA | - | NR | - |
| Microcephaly | NA | - | - | NA | - | NR | - |
| Neurological features |  |  |  |  |  |  |  |
| Delay in walking | + | + | + | NA | + | - | - |
| Speech delay | - | + | + | NA | + | + | - |
| ID | NR | - | NR | NA | Mild | NR | - |
| Behavioral anomalies | - | ADHD | - | NA | - | - | - |
| Seizures | - | - | - | NA | + | - | - |
| Brain abnormalities | NA | CC | NA | NA | NA | - | - |
| Eye |  |  |  |  |  |  |  |
| Ptosis | - | + | + | NA | - | + | + |
| Blepharophimosis | - | - | - | NA | - | + | - |
| Musculoskeletal anomalies |  |  |  |  |  |  |  |
| Hand | CD | - | NR | NA | - | - | - |
| Foot | - | - | NR | NA | SD | - | - |
| Growth |  |  |  |  |  |  |  |
| Feeding difficulty | + | + | - | NA | + | + | - |
| Short stature | + | - | - | NA | - | + | - |

Abbreviations: BD - brachydactyly, CC - corpus callosum abnormality, CD- camptodactyly, DSPF- down slanting palpebral fissures, M-male, F- female, NA - information not available, NR - information not reported, Y - years, M - months, + feature present; – feature absent.

**Supplemental Table 1D: Phenotype of patients in Group III**

| Group III | c.2497C>T (p.Arg833*) | | c.2915dup (p.Met973Asnfs*24) | c.2982 C>G (p. Tyr994*) | c.3298C>T (p.Arg1100*) | c.3459_3461del CTT (p.Phe1154del) | c.2420_2433del (p.Q807Lfs*27) | |
| --- | --- | --- | --- | --- | --- | --- | --- | --- |
| Pathogenic Variant  (inherited/*de novo*) | Inherited | *De novo* | *De novo* | *De novo* | *De novo* | *De novo* | *De novo* | |
| Age at diagnosis | 2Y | NR | 8Y | 10Y | 12Y | 20Y | 23Y | 28Y |
| Sex | M | NR | F | M | F | M | F | F |
| Craniofacial features |  |  |  |  |  |  |  |  |
| Flat facial profile | + | + | + | NR | + | - | - | - |
| DSPF | NR | - | + | + | + | + | - | - |
| Broad Nasal Root | NA | + | + | NR | + | - | + | + |
| Round face | - | + | - | + | + | - | - | - |
| Hypertelorism | + | + | + | NR | + | + | - | - |
| Head Circumference |  |  |  |  |  |  |  |  |
| Macrocephaly | - | NR | NR | NR | NR | + | + | + |
| Microcephaly | - | NR | NR | NR | NR | NR | - | - |
| Neurological features |  |  |  |  |  |  |  |  |
| Delay in walking | + | - | + | + | + | + | - | - |
| Speech delay | + | + | + | + | + | + | + | + |
| ID | Moderate to Severe | + | + | Moderate | + | Mild-Moderate | Mild | Mild |
| Behavioral anomalies | - | NR | NR | NR | NR | Autism and ADHD | ADHD | ADHD |
| Seizures | - | - | + | + | - | - | - | - |
| Brain abnormalities | - | NA | WM↓ | +* | - | CC | NA | NA |
| Eye |  |  |  |  |  |  |  |  |
| Ptosis | - | - | - | + | + | - | + | - |
| Blepharophimosis | - | - | - | + | + | - | - | - |
| Musculoskeletal anomalies |  |  |  |  |  |  |  |  |
| Hand | - | NR | NR | CD | NR | BD | - | - |
| Foot | - | NR | NR | - | NR | NR | - | - |
| Growth |  |  |  |  |  |  |  |  |
| Feeding difficulty | + | - | - | NR | + | NR | - | - |
| Short stature | + | NR | NR | - | NR | - | - | - |

Abbreviations: BD - brachydactyly, CC - corpus callosum abnormality, CD- camptodactyly, DSPF- down slanting palpebral fissures, M-male, F- female, NA - information not available, NR - information not reported, Y - years, M - months, + feature present; – feature absent, ↓ - decreased.

*Enlarged perivascular Virchow-Robin spaces.
